# Optimal row spacing improves the yield and quality of alfalfa cultivated in alpine areas of China

**DOI:** 10.3389/fpls.2026.1920341

**Published:** 2026-08-06

**Authors:** Longchao Xu, Manxi Guo, Yujia Gaoyan, Hairu Yang, Simeng Ding, Xiaoyang Zhou

**Affiliations:** College of Environment and Ecology, Taiyuan University of Technology, Taiyuan, China

**Keywords:** alfalfa, crude protein, RFV, row spacing, yield

## Abstract

Unprecedented pressures stemming from climate change and anthropogenic activities endanger grasslands, making well-designed forage cultivation practices essential for sustainable grassland management. We conducted a field experiment to determine the optimal planting density and cultivar to be used in the alpine region of Inner Mongolia. The experiment included 3 alfalfa cultivars cultivated at 4 different row spacings (20, 40, 60, and 80 cm) and we evaluated the responses of alfalfa yield and quality to these treatments. We found that the row spacing significantly affected the fresh and dry matteryield of alfalfa, and the effects differed by the cultivars. Specifically, cultivar polar bear (PB) and Longmu 806 (LM) cultivated with a 20 cm row spacing yielded significantly higher fresh and dry matter compared to the 60 cm and 80 cm treatments. Regarding the forage quality, the crude protein and crude fat of PB cultivated at 20 cm row spacing were more than those at 80 cm for both of two cuts, respectively, while the relative feed value (RFV) at 40 cm row spacing was 38.1% higher than that at 80 cm for the 2nd cut. On the other hand, the acidic detergent fiber and neutral detergent fiber at 80 cm row spacing were significantly higher than those of 60 and 20 cm for the 2nd cut. Regarding the LM cultivar, the ash and P contents in the 20 cm row spacing treatment were significantly higher than those at 80 cm. Different row spacing showed similar effects on the yield and quality indexes of the Gongnong (GN) cultivar. Principal component analysis indicated that quality factors, including crude protein and RFV, had the highest contribution rate, followed by yield factors and mineral element factors. We concluded that the PB should be the preferred cultivar for alfalfa planting in China’s northern alpine regions, with a recommended row spacing of 20–40 cm, followed by LM, with a recommended row spacing of 40–60 cm.

## 1 Introduction

Grasslands are under unprecedented pressure from climate change and human activities (Fang et al., 2018); therefore, sound forage cultivation practices are key to sustainable grassland management (Wang et al., 2026). Alfalfa (*Medicago sativa* L.) is a widely cultivated leguminous forage grass worldwide that is known as the “king of forages” due to its high nutritional value, resistance to mowing, high yield (Rogers et al., 2014) and high adaptability (Avci et al., 2010). Alfalfa cultivation is a widely used practice globally to improve soil fertility (Zhang et al., 2015) and provide high-quality forage for livestock (Shomar et al., 2010). Appropriate cultivation practices are necessary for the steady supply of alfalfa yields with high quality, and the selection of different row spacing distances is an important factor that directly affects both.

The microenvironment of the alfalfa canopy, including temperature, light, humidity, and CO_2_ concentration, can be improved by optimum row spacing, which could mitigate the competition between individuals in alfalfa populations regarding resource acquisition and survival (Wang et al., 2011, 2017), and then improve the yield and quality of alfalfa. However, the requirements for alfalfa seed amounts for sowing and row spacing also differ in regions with different climates and hydrothermal conditions. In the southeast of Europe, crude protein (CP) and dry matter (DM) yields of alfalfa sown at 20 cm row spacing were significantly higher than those at 50 and 100 cm (Stevovic et al., 2012). Avci et al. (2017) found that alfalfa forage yield at a 25 cm row spacing was remarkably higher than that with 50, 75, and 100 cm in North Africa, respectively. In China, Wang et al. (2020) and Nan et al. (2019) reported that alfalfa’s CP content and relative feeding value (RFV) were the highest at a row spacing of 20 cm. Lv et al. (2019) showed that in areas with saline-alkaline soils, an optimum row spacing of 15 cm could promote alfalfa growth, significantly increase its yield and CP content, and reduce neutral detergent fiber (NDF) and acidic detergent fiber (ADF) contents. Zhang et al. (2018) found that the yield and NDF and ADF contents decreased with a decrease in row spacing in the Huang-Huai-Hai region. However, it has also been reported that row spacing does not significantly affect alfalfa yield and quality indexes (Chocarro and Lloveras, 2015). Therefore, the selection of an optimal row spacing positively impacts the yield and quality of alfalfa while making full use of the available resources (Feng et al., 2022).

Hulunbuir is one of the important animal husbandry production bases in China. However, natural meadow have been increasingly degraded in recent years due to overgrazing and climate change (Zhu et al., 2022), and a feed shortage emerged. A rapid expansion of animal husbandry further intensified this feed shortage. Therefore, forage cultivation has begun to develop and has expanded in recent years, but the yield and quality of forage still failed to meet the requirements of industrial development (Ao et al., 2024). The feed source is still a bottleneck, limiting the development of grassland animal husbandry in China (Fang et al., 2018). Therefore, this study investigated the yield and quality of alfalfa of different cultivars under varying row spacing in the region. We tested whether the crop yields differed among the different row spacings and cultivars. We aimed to further develop the potential cultivation practices of alfalfa in such regions through a reasonable selection of suitable varieties and an optimal planting density, as well as provide a theoretical basis for attaining high alfalfa yields and high quality as a sustainable grassland management practice.

## 2 Materials and methods

### 2.1 Environment and soil properties

**Figure 1:**
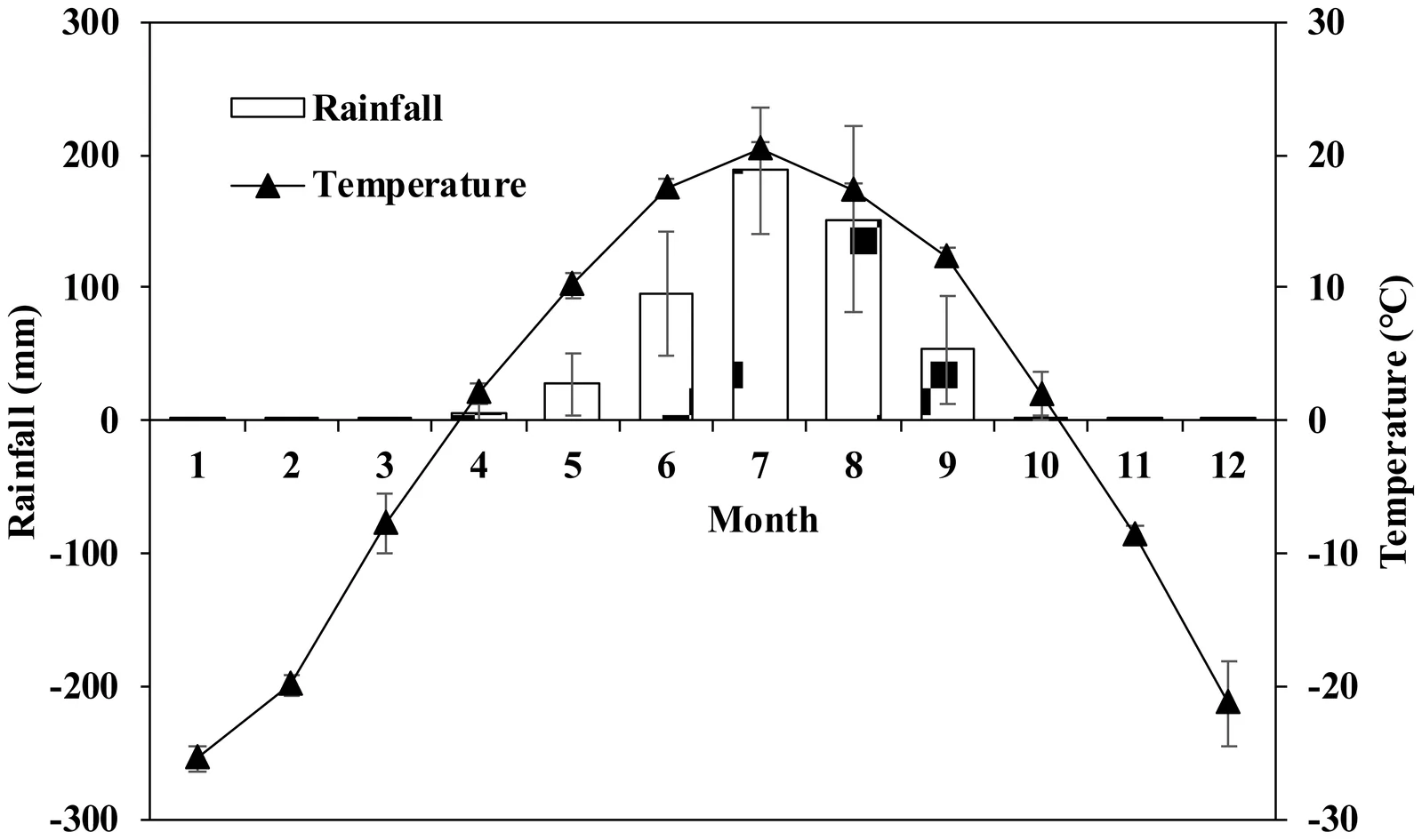
Annual temperature and rainfall in Hulun Buir Grass-based livestock husbandry experimental station.

A field experiment was set up at a trial field in the Hulunbuir Grass-based Livestock Husbandry Experimental Station, Institute of Botany, Chinese Academy of Science in Inner Mongolia, China (49°09′N; 119°75′E, 621 m elevation) in 2021. The experimental station was located at Bayantohai Town, Evenk Autonomous Banner, Hulunbuir City, which has a semi-arid continental monsoon climate in the middle temperate zone. The average annual temperature and frost-free season were -0.1°C and 112 days, and the average annual precipitation was 239.3 mm (between 2018 and 2020). The majority of precipitation in the region occurs in July and August (**Figure 1**).

The soil at the experimental site is chernozem soil and chestnut soil with a sandy-loamy texture. An analysis of an initial topsoil sample at a 0–20 cm depth revealed an organic matter content of 54.48 g·kg^-1^ and a pH value of 8.13. The average available N, P, and exchangeable K values were 227.36, 18.33, and 107.79 mg·kg^-1^, respectively.

### 2.2 Experimental design

In this experiment, 3 alfalfa cultivars were used as experimental materials, ‘Longmu 806’ (LM), ‘Gongnong 1’ (GN), and ‘Polar bear’ (PB), respectively. These cultivars are cold- and drought-tolerant cultivars introduced by the Institute of Botany, Chinese Academy of Sciences (Chen et al., 2012; Xu et al., 2013; Fang et al., 2022). The seed germination and field emergence rates of the 3 cultivars were above 91% and 86%, respectively. Four sowing row spacing distances (20, 40, 60, and 80 cm) were assessed for the 3 alfalfa cultivars, resulting in a total of 12 treatments. Each treatment was performed in 3 replicates, and a total of 36 plots with a net plot size of 4 m×5 m (20 m^2^) was set up. In this experiment, the seeds were sown by manual strip sowing in June 2020, and the sowing rate was 15 kg·ha^-1^. Alfalfa was harvested twice throughout the growing season, and the mowing was carried out in July and September 2021, respectively.

The fertilizer applied in this experiment was diammonium phosphate, potassium chloride, and urea mixed in a ratio of 3:2:1, with 450 kg·ha^-1^ applied to all treatments. After each mowing, 150 kg·ha^-1^ of diammonium phosphate was applied to all treatments. Standard procedures were followed for other agronomic measures, such as weeding and watering.

### 2.3 Plant trait analysis and measurements

Different alfalfa traits were determined during the early flowering stage. The height of 15 randomly selected alfalfa plants was measured in each plot. Fresh alfalfa was weighed after mowing with 5 cm of stubble from 0.6 m × 1 m quadrats for each plot, and the values were extrapolated to estimate fresh alfalfa yield per hectare. Each plot was replicated 4 times. The fresh alfalfa was dried in an oven at 65°C, and the DM yield was measured after reaching constant weight to determine the DM yield per hectare. The dry-to-fresh ratio was calculated using fresh alfalfa yield and the corresponding DM yield.

About 500g of fresh-cut alfalfa was randomly selected in each plot, and after drying at 105°C for 0.5 h in an oven, it was dried to constant weight at a constant temperature of 65°C. Then, the quality traits of alfalfa were assessed following grinding and sieving through a 60-mesh sieve. The quality of alfalfa is primarily categorized into two components: nutritional quality index and feed quality index. Nutrient content indicators included crude protein (CP), crude fat (CF), neutral detergent fiber (NDF), acidic detergent fiber (ADF), lignin, ash, calcium (Ca), phosphorus(P), magnesium (Mg) and potassium (K). Feed quality evaluation indicators included relative feed value (RFV), total digestible nutrients (TDN), net lactation energy (NLE), maintenance net energy (MNE), and net energy for growth (NEG). The RFV (Rohweder et al., 1978) was calculated based on the NDF and ADF and was calculated as:


$$RFV=\frac{120\times \left(88.9-0.779\times ADF\right)}{1.29\times NDF}$$

Alfalfa quality indicators were determined using near-infrared reflectance spectroscopy (NIRS). NIRS is a simple, rapid, and efficient qualitative and quantitative analysis technique widely used in forage quality testing, including nutrient composition indicators and feeding value assessment. In this experiment, the near-infrared rapid detection model for alfalfa from Cumberland Valley Analytical Services (CVAS) was used as the basis for data analysis.

The topsoil (0-20cm) was initially assessed for 5 chemical properties. Soil pH was measured by a pH meter with a soil-to-water ratio of 1:2.5. The potassium dichromate external heating method was used for soil organic matter determination. Alkali hydrolyzable nitrogen was determined by the alkaline hydrolysis diffusion method. The available phosphorus was determined by the Olsen method. Available potassium was extracted by ammonium acetate and determined using a flame photometer (Bao, 2000).

### 2.4 Statistical analyses

SPSS Statistics 25 was used to perform one-way analysis of variance, multivariate analysis of variance, and PCA on the data. Differences between means were assessed using the LSD test. The figures were drawn using R (R 4.4.2).

## 3 Results

### 3.1 Effects of different row spacings and cultivars on alfalfa yield

According to the analysis of variance (**Supplementary Table 1**), the row spacing had a significant effect on the fresh and dry matter (DM) yield of alfalfa (*p<* 0.001) but did not significantly affect alfalfa plant height (PH) and plant dry-to-fresh ratio. Different cuts exhibited significant differences in PH, fresh yield and DM, and dry-fresh ratio of alfalfa (*p* < 0.05), and these indexes did not differ by cultivars (**Table 1**). The interaction effect of alfalfa cultivars and cuts significantly affected the dry-fresh ratio of alfalfa (*p* < 0.05), but had no significant effect on other indicators.

**Table 1:** Analysis of variance of alfalfa plant height and yield indicators.

Factor	Plant height	Fresh yield	DM yield	DM/fresh yield
Cultivar (cv)	NS	NS	NS	NS
Row spacing (Rs)	NS	***	***	NS
Cuts (cs)	***	***	**	*
cv×Rs	NS	NS	NS	NS
cv×cs	NS	NS	NS	*
Rs×cs	NS	NS	NS	NS
cv×Rs×cs	NS	NS	NS	NS

×Indicates interactions between the factors. *, ** and *** indicate significant difference at the level of 0.05, 001 and 0.001, respectively. NS indicates no significant different. The same as below.

**Figure 2:**
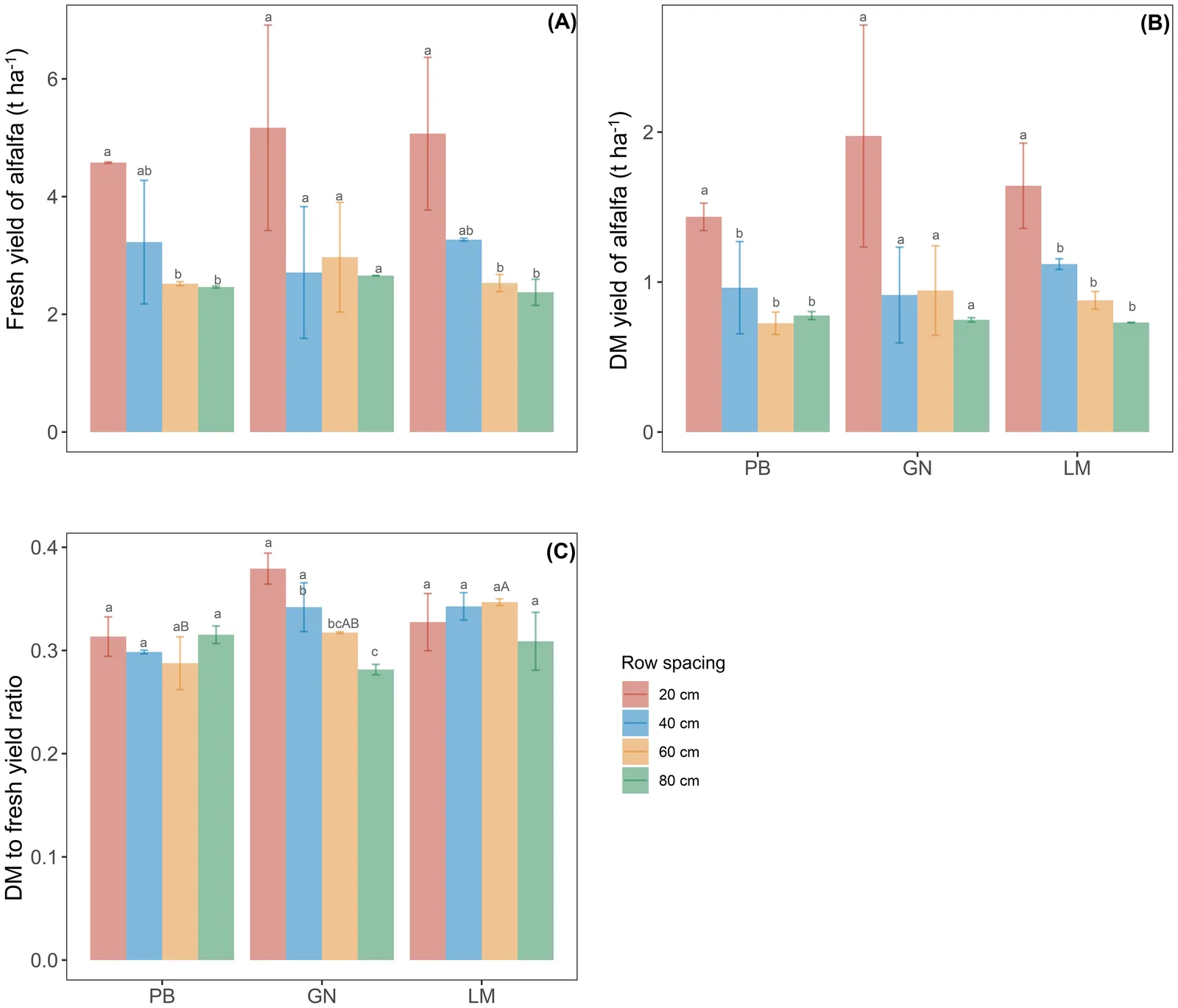
Effects of different row spacings on fresh yield **(A)**, DM yield **(B)** and the ratio of DM and fresh weight **(C)** of alfalfa. Different lowercase and uppercase letters are statistically different at different row spacing treatments and different cultivar treatments, respectively (*P* < 0.05). PB, Polar bear; GN, Gongnong 1; LM, Longmu 806; DM, dry matter yield. Error bars represent standard error.

Different row spacing treatments significantly affected the fresh yield of all cultivars (*p* < 0.05; **Figure 2**). However, there was no significant difference in alfalfa fresh yield between the different cultivars. Thus, non-significant effect of the genotype was observed (**Figure 2**, *p* > 0.05). In both PB and LM alfalfa cultivars, both the fresh yield and DM at 20 cm spacing were significantly higher than that at 60 and 80 cm, respectively (*p* < 0.05; **Figure 2**). In the GN cultivar, both fresh and DM yields were not affected by row spacings, respectively. Overall, it was apparent that fresh and DM yields of alfalfa were greater, with narrower rows for all cultivars (**Figure 2**). The fresh to DM yield ratio of GN at a row spacing of 20 cm was higher than 60 and 80 cm, while 40 cm spacing resulted in a higher yield than 80 cm. (*p* < 0.05; **Figure 2**). Moreover, the fresh-to-DM yield ratio of LM was higher than that of PB in the 60 cm row spacing (*p<* 0.05).

### 3.2 Effects of different row spacings and cultivars on alfalfa quality

The different alfalfa cultivars significantly affected the contents of lignin, ash, Ca, and K (*p* < 0.05), while different row spacing distances significantly affected the contents of CP, ADF, NDF, ash, Ca, and K in alfalfa (*p* < 0.05; **Table 2**). Moreover, the different cuts exhibited significant differences in the contents of CP, ADF, NDF, lignin, Ca, P, Mg, and K (*p* < 0.05). There was a significant interaction between cultivars and cuts on ash content (*p* < 0.05), and a significant interaction between cultivars, row spacing, and cuts on P content (*p* < 0.05; **Supplementary Table 2**).

**Table 2:** Analysis of variance of alfalfa nutritional quality indicators.

Factor	CP	CF	ADF	NDF	Lignin	Ash	Ca	P	Mg	K
Cultivar (cv)	NS	NS	NS	NS	*	**	*	NS	NS	*
Row spacing (Rs)	*	NS	*	*	NS	*	*	NS	NS	*
Cuts (cs)	***	***	***	***	***	NS	***	***	***	***
cv×Rs	NS	NS	NS	NS	NS	NS	NS	NS	NS	NS
cv×cs	NS	NS	NS	NS	NS	*	NS	NS	NS	NS
Rs×cs	NS	NS	NS	NS	NS	NS	NS	NS	NS	NS
cv×Rs×cs	NS	NS	NS	NS	NS	NS	NS	*	NS	NS

CP, CF, ADF and NDF indicate crude protein, crude fat, acidic detergent fibers and neutral detergent fibers, respectively.

**Figure 3:**
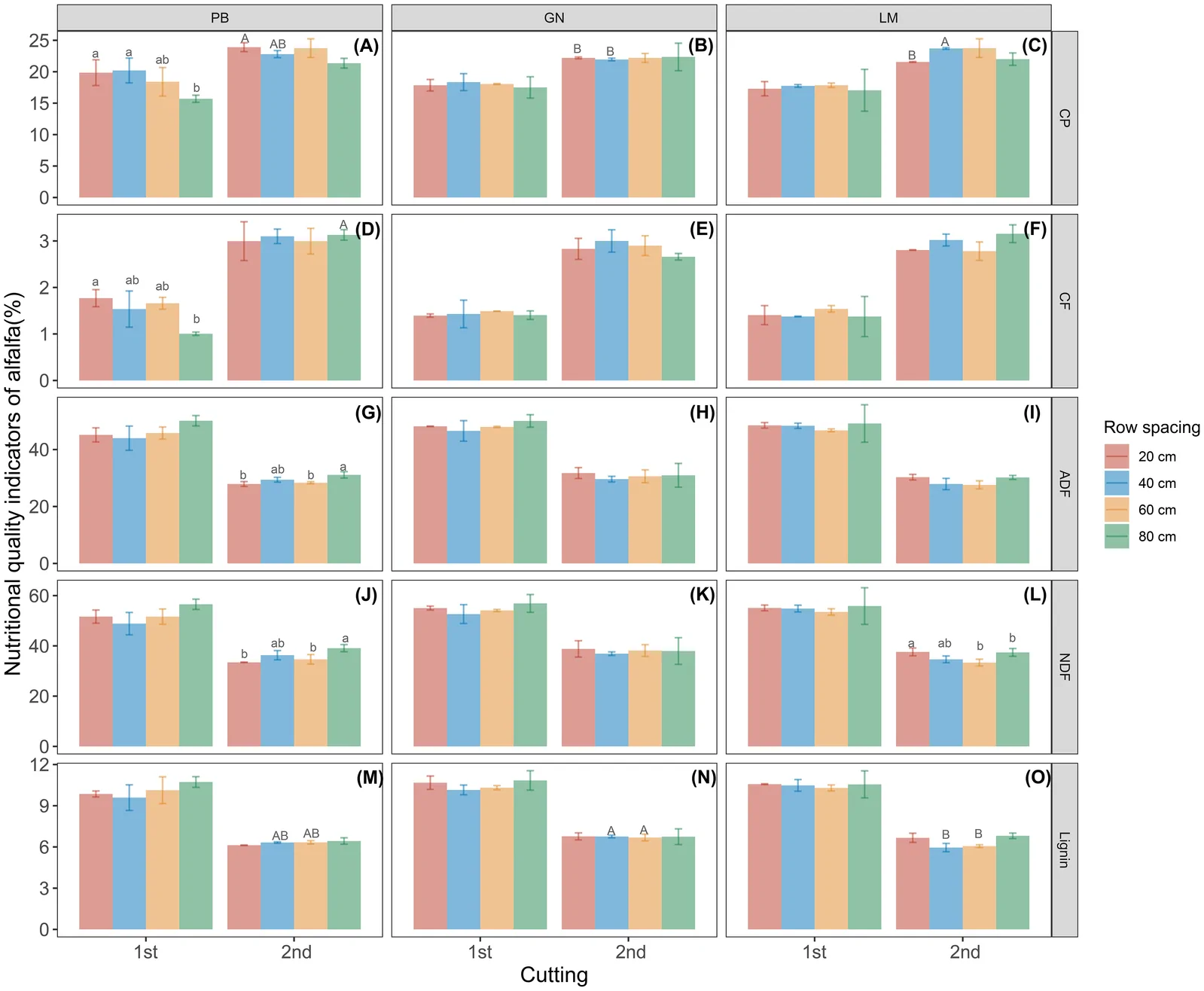
Effects of different row spacing and cultivar on nutritional quality indicators of alfalfa. **(A, D, G, J, M)** are for CP, CF, ADF, NDF and ligmin in PB, respectively; **(B, E, H, K, N)** are for CP, CF, ADF, NDF and ligmin in GN, respectively; **(C, F, I, L, O)** are for CP, CF, ADF, NDF and ligmin in LM, respectively. Different lowercase and uppercase letters are statistically different at different row spacing treatments and different cultivar treatments, respectively (*P* < 0.05). CP, crude protein; CF, crude fat; ADF, acidic detergent fiber; NDF, neutral detergent fiber. See **Figure 2** for abbreviation of different cultivars. Error bars represent standard error.

For the PB cultivar, CP content of the 1^st^ cut at 20 and 40 cm row spacing was 19.85 and 20.20%, respectively, which were 26.5 and 25.2% higher than that at the 80 cm row spacing treatment (*p<* 0.05; **Figure 3**). The CF content at 20 and 60 cm row spacing was 19.85 and 18.40%, respectively, higher compared to that at 80 cm (*p<* 0.05; **Figure 3**). ADF and NDF contents of the 2^nd^ cut at 80 cm row spacing were 31.10 and 39.10%, respectively, significantly higher than those at 60 and 20 cm (*p<* 0.05). For the LM cultivar, the NDF content of the 1^st^ cut at 20 cm row spacing was significantly higher than that at 60 cm (*p* < 0.05; **Figure 3**). The CP of the 2^nd^ cut in the PB cultivar alfalfa was significantly higher compared to the other cultivars at a row spacing of 20 cm. The CP of alfalfa in the LM cultivar was significantly higher than that of the GN cultivar in the 40 cm row spacing treatment. The lignin content of GN for the 2^nd^ cut at the 40 cm and 60 cm row spacing was 6.76 and 6.69%, respectively, higher than the values measured in LM (**Figure 3**).

**Figure 4:**
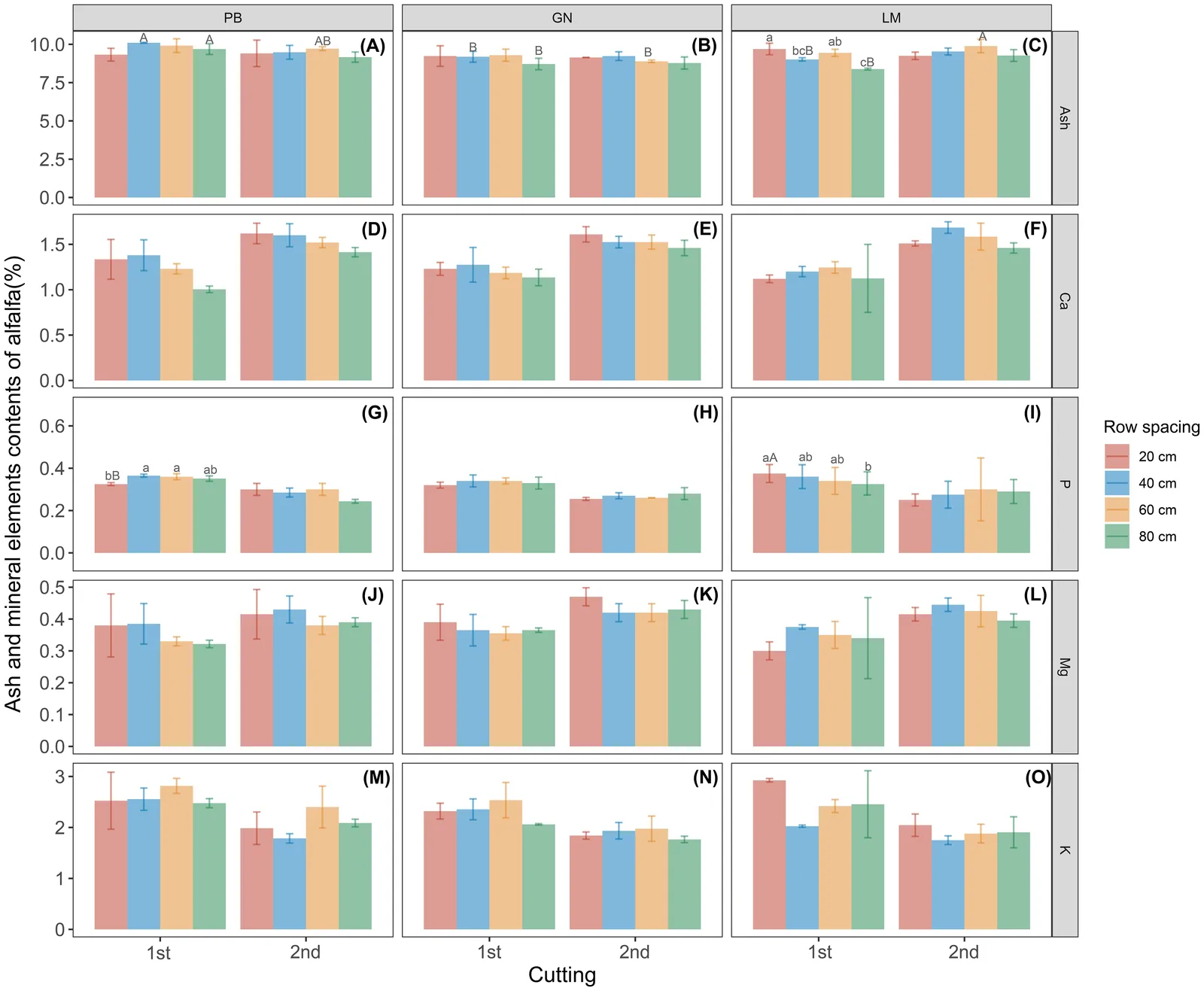
Effects of different row spacing and cultivar on ash and mineral elements contents of alfalfa. **(A, D, G, J, M)** are for ash, Ca, P, Mg and K in PB, respectively; **(B, E, H, K, N)** are for ash, Ca, P, Mg and K in GN, respectively; **(C, F, I, L, O)** are for ash, Ca, P, Mg and K in LM, respectively. Different lowercase and uppercase letters are statistically different at different row spacing treatment and different cultivar treatment, respectively (*P* < 0.05). Ca, calcium; P, phosphorus; Mg, magnesium; K, potassium. See **Figure 2** for abbreviation of different cultivars. Error bars represent standard error.

The P contents of the 1^st^ cut in the PB cultivar under the 40 and 60 cm row spacing treatments were 0.37 and 0.36%, respectively, significantly higher than that at 20 cm (*p<* 0.05; **Figure 4**). The alfalfa ash content of the 1^st^ cut in the LM cultivar at 20 cm row spacing was 9.70%, significantly higher than that at 40 and 80 cm (*p<* 0.05). The P content of the 1^st^ cut in the LM cultivar at 20 cm row spacing was significantly higher than at 80 cm (*p<* 0.05; **Figure 4**). The ash contents of alfalfa stubble were the highest in the PB cultivar at a row spacing of 40 cm and 80 cm, reaching 10.10 and 9.69%, respectively, significantly higher compared to the GN and LM cultivars at the corresponding row spacing treatments (*p* < 0.05; **Figure 4**). The LM cultivar had the highest ash content of the 2^nd^ cut in the 60 cm row spacing treatment, significantly higher than PB and GN cultivars (*p* < 0.05). There was no significant difference in Ca, Mg, and K contents in alfalfa among different cultivars and row spacing treatments (*p* > 0.05; **Figure 4**).

**Figure 5:**
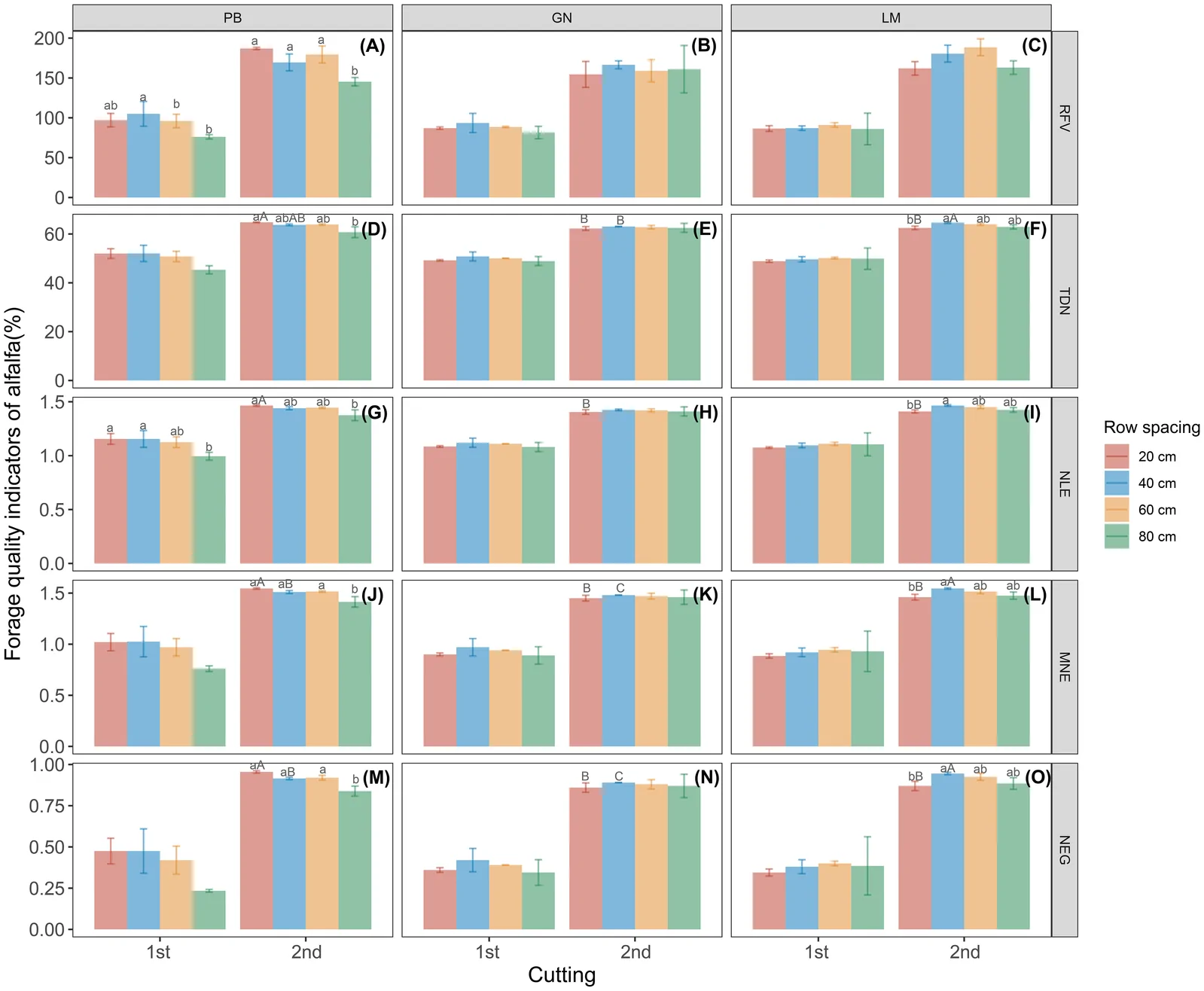
Effects of different row spacing and cultivar on forage quality indicators of alfalfa. **(A, D, G, J, M)** are for RFV, TDN, NLE, MNE and NEG in PB, respectively; **(B, E, H, K, N)** are for RFV, TDN, NLE, MNE and NEG in GN, respectively; **(C, F, I, L, O)** are for RFV, TDN, NLE, MNE and NEG in LM, respectively. Different lowercase and uppercase letters are statistically different at different row spacing treatment and different cultivar treatment, respectively (*P* < 0.05). RFV, relative feed value. TDN, total digestible nutrients; NLE, net lactation energy; MNE, maintenance net energy; NEG, net energy for growth. See **Figure 2** for abbreviation of different cultivars. Error bars represent standard error.

In the PB cultivar, alfalfa at a 40 cm row spacing had a significantly higher RFV (97.0) than at an 80 cm row spacing. The RFV of alfalfa was 187.0, 169.5, and 179.5 at a row spacing of 20, 40, and 60 cm, respectively, significantly higher compared to that of 80 cm (*p* < 0.05; **Table 3**). The TDN content at a 20 cm row spacing was 64.8%, significantly higher than that at 80 cm. The NEL at a 20 cm row spacing in the1st and 2^nd^ cuts were 1.16 and 1.47 Mcal·kg^-1^, respectively, significantly higher compared to the 80 cm row spacing treatment (*p* < 0.05). The MNE and NEG of alfalfa at a row spacing of 20, 40, and 60 cm were significantly higher than those at 80 cm (*p<* 0.05; **Figure 5**). Regarding the LM cultivar, the contents of TDN, NEL, MNE, and NEG at a 40 cm row spacing were 64.7%, 1.47, 1.55, and 0.95 Mcal·kg^-1^, respectively, and were significantly higher than those at 20 cm (**Figure 5**). Moreover, no significant differences were observed between the 60 cm and 80 cm row spacing treatments and other treatments.

**Table 3:** Analysis of variance of alfalfa forage quality indicators.

Factor	RFV	TDN	NEL	MNE	NEG
Cultivar (cv)	NS	NS	NS	NS	NS
Row spacing (Rs)	*	*	*	*	*
Cuts (cs)	***	***	***	***	***
cv×Rs	NS	*	*	NS	NS
cv×cs	NS	NS	NS	NS	NS
Rs×cs	NS	NS	NS	NS	NS
cv×Rs×cs	NS	NS	NS	NS	NS

RFV, TDN, NEL, MEE and NEG indicate relative feeding value, total digestible nutrients, net energy lactation, maintain net energy and net energy for growth, respectively.

The alfalfa feed quality indicators were significantly affected by the row spacing (*p* < 0.05) and also exhibited significant differences between alfalfa cuts (*p<* 0.001). At the same time, different varieties had no significant effect on the feed quality indexes (*p* > 0.05). Cultivars and row spacing significantly affected TDN and LEN (*p<* 0.05). However, they had no significant effect on RFV, MNE, and NEG (*p* > 0.05; **Table 3**). Finally, no significant interactions were observed between cultivars and cuts, row spacing and cuts, or any of the three factors that affected alfalfa feed quality indexes (**Table 3**).

In the PB cultivar, the RFV of 40 cm row spacing for 1^st^ cut was 38.1% higher than that in 80 cm (*p<* 0.05), and RFV of 20, 40 and 60 cm row spacings in 2^nd^ cut were 28.7, 16.7 and 23.6% more than that in 80 cm (**Figure 5**). The PB cultivar had significantly higher TDN, NEL, MNE, and NEG than the other two cultivars in the 20 cm row spacing treatment for the 1^st^ cut (*p<* 0.05). At a 40 cm row spacing, the TDN content of the 2^nd^ cut in the alfalfa LM cultivar was significantly higher than that of GN (*p<* 0.05), while MNE and NEG contents in LM were significantly higher than those in PB (**Figure 5**). Their contents were significantly higher in LM and PB than in GN (*p<* 0.05).

### 3.3 Principal component analysis including all alfalfa yield and quality indicators

Principal component analysis (PCA) was implemented to reduce the data dimensionality to eliminate the influence of overlapping information. When assessing the effects of the different row spacing and cultivar treatments, we should not only consider the advantages and disadvantages of one or several indicators but should comprehensively evaluate them as a whole. In our dataset, the KOM value was 0.613, and the Sig of Bartlett test was 0.000, indicating that PCA could be implemented in the data. Based on eigen value scores greater than 1, 4 principal components could be extracted, and the cumulative variance contribution rate is 87.987%, indicating that these 4 principal components could explain 87.987% of the overall variance (**Supplementary Table 1** in appendix).

The eigen vectors and loading values corresponding to the principal components are shown in appendix (**Supplementary Table 2**). The first principal component mainly explained the variance in NEG(X_14_), MNE(X_13_), RFV(X_10_), CP(X_5_), TND(X_11_), ADF(X_7_), NDF(X_8_) and NEL(X_12_) which had large loading values and coefficients of 0.982, 0.975, 0.958, 0.952, 0.947, -0.944, -0.939 and 0.932, respectively. The above indicators correspond to nutritional quality and feed quality indicators. Thus, PC1 was informative regarding variance in quality factors. The second principal component mainly explained variance in K(X_19_), P(X_17_), Mg(X_18_), and ash (X_15_), with coefficients of 0.873, 0.784, -0.759, and 0.682, respectively. Thus, PC2 was informative regarding variance in mineral element factors. The third principal component explained variance in dry weight (X_3_) and fresh weight (X_2_); their loading values were 0.973 and 0.932, respectively, and were informative regarding variance in yield factors. The fourth principal component explained the variance in the dry-fresh ratio (X_4_), and its loading value was -0.544.

The model was used to calculate and rank the comprehensive scores of different varieties and seeding row spacing treatments (**Table 4**). The evaluation score for the quality factor (Y_1_) indicated that the PB alfalfa cultivar exhibited a high-quality factor score at row spacings of 20–60 cm. In comparison, the LM cultivar demonstrated a high-quality factor score at 40 and 60 cm. Regarding the evaluation score of mineral element factors (Y_2_), the scores of PB at 40 and 60 cm and of LM at 20 cm were higher. The alfalfa yield factor (Y_3_) scores of the PB, GN, and LM cultivars were greater at 20 cm compared to other row spacing treatments. The following order was established based on the comprehensive scores for the various cultivars: PB > LM > GN. The PB cultivar had a high score at a row spacing of 20, 40, and 60 cm, and the LM cultivar at 60 and 40 cm. Collectively, the results showed that the PB cultivar was more suitable for alfalfa planting in Hulunbuir than the other two cultivars.

**Table 4:** Comprehensive rankings and scores of different cultivar and row spacing.

Cultivar	Row spacing	Y1	Y2	Y3	Y4	Y	Ranking
PB	20 cm	4.327	0.444	1.892	1.333	2.765	2
	40 cm	4.257	1.089	0.064	0.485	2.784	1
	60 cm	2.551	3.160	-1.095	-0.488	2.102	3
	80 cm	-6.173	0.905	-1.267	-1.630	-3.610	12
GN	20 cm	-2.063	-2.881	3.330	-0.250	-1.702	10
	40 cm	0.831	-0.937	-0.676	-0.791	0.296	6
	60 cm	-0.717	-0.736	-0.537	0.085	-0.586	7
	80 cm	-2.861	-1.081	-1.858	2.231	-1.957	11
LM	20 cm	-2.630	1.336	2.052	-0.305	-1.283	9
	40 cm	1.944	-1.659	-0.177	-0.472	0.850	5
	60 cm	2.009	0.580	-0.100	-0.907	1.309	4
	80 cm	-1.475	-0.219	-1.628	0.709	-0.969	8

Y indicates composite score.

## 4 Discussion

### 4.1 Effects of different row spacings on alfalfa yield

Appropriate row spacing or seeding density is one of the critical factors to effectively exploit the high yield potential of alfalfa (Lv et al., 2019; Wang et al., 2022). Chocarro and Lloveras (2015) found that the DM yield of alfalfa tended to decrease with the increase in row spacing (20–60 cm) in Lleida, Spain, but no significant differences could be established. Avci et al. (2017) conducted alfalfa experiments with different row spacing in the Adana area of Turkey. They found that the three-year average yield of alfalfa at 25 cm row spacing was significantly higher compared to that at 75 and 100 cm. However, there was no significant difference between alfalfa seed yield with different row spacing. Stevovic et al. (2012) carried out an alfalfa experiment in Zajecar, Serbia. They found that the DM yield of alfalfa at a row spacing of 20 cm was significantly higher than that at 50 cm, and both were significantly higher than 100 cm. The leaf and stem number were the highest at 20 cm row spacing, significantly higher than that at 100 cm. The above studies can all conclude that suitable plant density can significantly increase alfalfa yield and improve alfalfa growth performance, and this optimum fall between 20 and 25 cm. In this study, the 20 cm row spacing resulted in a significantly higher yield in the PB and LM cultivars compared to 60 and 80 cm (*p<* 0.05). This is consistent with the results of Meng et al. (2019), which found that alfalfa yield at a 15 and 30 cm row spacing was significantly higher than that at 5 and 60 cm. In this study, the fresh-to-dry yield ratio of the GN cultivar at 20 cm was significantly higher than that at 60 cm and 80 cm (*p<* 0.05). These results indicated that narrow row spacing (20 cm) was the most beneficial planting method for increasing alfalfa yield in Hulunbuir. Planting density can promote the efficient use of environmental resources such as light, heat, water, and fertilizer per unit area, thereby increasing the accumulation of dry matter, obtaining higher yield (Wang et al., 2022), and increasing the ratio of dry to fresh weight. Under the same overall sowing rate, the alfalfa yield at the 80 cm row spacing treatment was only 38-54% of at 20 cm row spacing, primarily attributable to the excessive seeds sown per row due to the increased row spacing. This, in turn, caused poor ventilation and reduced light absorption by the plant population, which affected the normal photosynthesis levels. As a result, the alfalfa population exhibited a self-thinning phenomenon (Meng et al., 2019), resulting in a subsequent decline in yield.

Many studies have shown that alfalfa yield at the 1^st^ cut was the highest under continuous mowing. Then the yield of each cut showed a decreasing trend with the increase in the number of cuts (Zhao et al., 2016), and the contribution rate of the 1st cut to the total yield was greater than that of other cuts (Wang et al., 2009). Consistent with these, in this study, the fresh and DM yield of all alfalfa cultivars was greater in the 1^st^ than the 2^nd^ cut, indicating that the 1^st^ cut was significant for the total annual yield (Xiao et al., 2017). We also found that the plant height at the 1^st^ cut was significantly higher than that at the 2^nd^ cut (*p<* 0.05). Therefore, good field management before the 1^st^ cut of alfalfa is the key to achieving yield increase. The 2^nd^ cut of alfalfa grows during the high temperature and precipitation season in the central part of Inner Mongolia. Consequently, the second cut of alfalfa has a significant potential for yield improvement under proper field management practices during this period (Chen et al., 2014).

### 4.2 Effects of different row spacings and cultivars on alfalfa quality

Forage quality can directly determine its feeding and commercial value. CP, NDF, ADF, RFV, CF, ash, and mineral elements are important components in measuring alfalfa’s nutritional and feeding value (Yang, 2010). Stevovic et al. (2012) analyzed the quality of alfalfa and found that the CP and ash content reached the highest values at a row spacing of 20 cm (19.43 and 7.14%, respectively), which was significantly higher than that at 100 cm. Moreover, the crude fiber content of alfalfa in the 20 cm row spacing treatment was significantly lower than that at 50 and 100 cm. Celebi et al. (2010) also reported that the CP content reached its highest values at a row spacing of 20 cm, followed by 30 and 40 cm. However, some studies have found that row spacing or planting density does not significantly affect the CP content of alfalfa or other crops (Lloveras et al., 2008; Ramanjaneyulu et al., 2018). In this study, the CP and CF contents in alfalfa were the highest in the PB cultivars at a row spacing of 20 cm, significantly higher than that at 80 cm (*p* < 0.05). This phenomenon may be due to that planting density per unit distance increase with the decrease in row spacing. This weakens the competition for light between plant individuals, and promotes cell elongation (Zhang et al., 2016a), the rapid growth of stems (Wang et al., 2015), and an increase in the stem-to-leaf ratio. Consequently, the quality of the alfalfa is reduced, as evidenced by a decrease in the CP content. However, in this study, there was no significant difference in the CP and CF contents of alfalfa in the GN and LM cultivars between different row spacing. It can be, therefore, concluded that under the same external environment and planting scheme, the response of quality indicators such as alfalfa CP to the row spacing of different cultivars is different.

Alfalfa NDF and ADF are international indicators to evaluate its feeding potential and digestibility. Low NDF content in alfalfa indicates good quality, while ADF content is negatively correlated with digestibility, with increased ADF content reducing digestibility in livestock (Katić et al., 2009). RFV is a comprehensive reflection of NDF and ADF that can be used to predict feed intake and energy value, with higher values reflecting a higher nutritional value of the roughage (Chen et al., 2015). In this study, the NDF and ANF contents of alfalfa in the PB cultivar were the highest at a row spacing of 80 cm, significantly higher than those at 20 and 60 cm. The RFV of the 1^st^ cut of alfalfa reached its highest value at 40 cm, significantly higher than that of 80 cm. Moreover, the RFV of the 2^nd^ cut was significantly higher at 20, 40, and 60 cm than at 80 cm. This is mainly because the number of alfalfa shoots per unit area increased with narrow row spacing, leading to the inhibition of alfalfa growth and development due to the reduced growth space. As a result, slenderer stem and the greatest proportion of leaves appeared commonly (Lamb et al., 2014; Zhang et al., 2016b), resulting in high CP, CF contents and high RFV, and low ADF and NDF contents (Grant et al., 2014; Lv et al., 2019). In addition, the lignin content increased with the increase in row spacing (**Table 4**), indicating that the digestibility of alfalfa decreased with row spacing increase (Volenec et al., 1987). In conclusion, previous studies and our study suggest that reducing the row spacing is advantageous for enhancing the quality of alfalfa.

The important indicators reflecting the nutritional quality characteristics of alfalfa are crude CP, CF, NDF, ADF, and RFV (Wang et al., 2017). Alfalfa products with CP > 19%, NDF< 40%, ADF< 31%, and RFV > 155% are considered of high quality in the international grass products market (Kazemi et al., 2012). Across all treatments in this experiment, the 2^nd^ cut alfalfa was classified as high-quality alfalfa based on the CP, NDF, ANF, and RFV values. Although the yield of 2^nd^ cut alfalfa was significantly lower than that of 1^st^ cut, it had higher economic value and high quality, so it was necessary to comprehensively evaluate alfalfa quality, including multiple indicators. The PCA overcomes the drawbacks of relying on the evaluation of a single trait and can objectively reflect the comprehensive performance of many traits in the production performance (Xu et al., 2014; Wang et al., 2022). In this study, we combined plant height, yield, and quality indicators to comprehensively evaluate and rank alfalfa yield and quality traits by PCA to avoid using only a single index when determining the optimal selection regarding the cultivar and row spacing.

Nevertheless, this study has several limitations. The experiment was conducted at a single site over a limited period, which may restrict the generalizability of the findings to other alpine or semi-arid regions with varying soil types and microclimates. Additionally, long-term effects on soil health, biodiversity, and persistence of alfalfa stands under different planting densities remain unclear and require multi-year, multi-location trials. Economic feasibility and potential interactions with other management practices (e.g., irrigation, fertilization) were not fully assessed in the current study.

## 5 Conclusions

Forage cultivation practices are essential for sustainable grassland management, especially as grasslands worldwide come under unprecedented pressure from climate change and human activities. To achieve productive cultivation of alfalfa with high quality in the alpine regions of China, the selection of suitable cultivars and reasonable row spacing are of great importance. Based on a comprehensive evaluation of alfalfa yield and quality in this study, we conclude that the “Polar bear” cultivar is recommended as the preferred choice for alfalfa planting in the Hulunbuir area of China, at a row spacing of 40 or 20 cm, followed by the LM cultivar, with a recommended row spacing of 60 or 40 cm.

## Data Availability

The raw data supporting the conclusions of this article will be made available by the authors, without undue reservation.
