# Supplementary material for: Optimal row spacing improves the yield and quality of alfalfa cultivated in alpine areas of China
Source: Front Plant Sci. 2026 Aug 6;17:1920341. doi: 10.3389/fpls.2026.1920341 (PMC13490176; doi:10.3389/fpls.2026.1920341)
Supplement: Supplementary file 1 [file Table1.docx]

Supplementary Material

# Supplementary Tables

Table S1 Eigen value of principal components

| Principal component | Variance of initial eigen values | | |
| --- | --- | --- | --- |
|  | Eigenvalue | Variance contribution (%) | Accumulative contribution (%) |
| 1 | 10.094 | 53.126 | 53.126 |
| 2 | 3.161 | 16.638 | 69.763 |
| 3 | 2.416 | 12.718 | 82.481 |
| 4 | 1.046 | 5.506 | 87.987 |
| 5 | 0.729 | 3.839 | 91.826 |
| 6 | 0.470 | 2.473 | 94.298 |
| 7 | 0.398 | 2.093 | 96.392 |
| 8 | 0.240 | 1.261 | 97.653 |
| 9 | 0.200 | 1.055 | 98.708 |
| 10 | 0.089 | 0.469 | 99.177 |
| 11 | 0.065 | 0.343 | 99.520 |
| 12 | 0.049 | 0.260 | 99.780 |
| 13 | 0.022 | 0.114 | 99.894 |
| 14 | 0.009 | 0.049 | 99.943 |
| 15 | 0.005 | 0.024 | 99.968 |
| 16 | 0.003 | 0.018 | 99.985 |
| 17 | 0.002 | 0.011 | 99.996 |
| 18 | 0.001 | 0.003 | 99.999 |
| 19 | 0.000 | 0.001 | 100.000 |
