# Supplementary material for: Optimal row spacing improves the yield and quality of alfalfa cultivated in alpine areas of China
Source: Front Plant Sci. 2026 Aug 6;17:1920341. doi: 10.3389/fpls.2026.1920341 (PMC13490176; doi:10.3389/fpls.2026.1920341)
Supplement: Supplementary file 2 [file Table2.docx]

Supplementary Material

# Supplementary Tables

Table S2 The eigenvectors and load matrix corresponding to the principal components

| Item | Principal component 1 (Y_1_) | | Principal component 2 (Y_2_) | | Principal component 3 (Y_3_) | | Principal component 4 (Y_4_) | |
| --- | --- | --- | --- | --- | --- | --- | --- | --- |
|  | Feature vetor | Load | Feature vetor | Load | Feature vetor | Load | Feature vetor | Load |
| PH(X_1_) | -0.161 | -0.513 | 0.272 | 0.484 | 0.214 | 0.332 | 0.377 | 0.385 |
| Fresh yield (X_2_) | -0.046 | -0.146 | 0.013 | 0.024 | 0.600 | 0.932 | 0.217 | 0.222 |
| DM yield (X_3_) | -0.049 | -0.154 | -0.045 | -0.081 | 0.626 | 0.973 | 0.062 | 0.063 |
| DM / Fresh (X_4_) | -0.006 | -0.019 | -0.247 | -0.440 | 0.371 | 0.576 | -0.532 | -0.544 |
| CP (X_5_) | 0.300 | 0.952 | 0.024 | 0.042 | 0.041 | 0.063 | 0.116 | 0.118 |
| CF (X_6_) | 0.205 | 0.652 | 0.174 | 0.310 | 0.072 | 0.113 | -0.016 | -0.016 |
| ADF (X_7_) | -0.296 | -0.939 | -0.020 | -0.036 | 0.022 | 0.035 | 0.213 | 0.218 |
| NDF (X_8_) | -0.297 | -0.944 | -0.051 | -0.090 | 0.037 | 0.058 | 0.151 | 0.154 |
| Lignin (X_9_) | -0.269 | -0.854 | -0.102 | -0.181 | -0.014 | -0.022 | 0.196 | 0.201 |
| RFV (X_10_) | 0.302 | 0.958 | 0.071 | 0.126 | -0.017 | -0.026 | -0.013 | -0.014 |
| TND (X_11_) | 0.298 | 0.947 | -0.010 | -0.017 | 0.048 | 0.074 | 0.200 | 0.204 |
| NEL (X_12_) | 0.294 | 0.932 | -0.015 | -0.026 | 0.048 | 0.075 | 0.248 | 0.254 |
| MNE (X_13_) | 0.307 | 0.975 | -0.012 | -0.021 | 0.021 | 0.033 | 0.142 | 0.146 |
| NEG (X_14_) | 0.309 | 0.982 | -0.013 | -0.023 | 0.025 | 0.038 | 0.126 | 0.129 |
| Ash (X_15_) | 0.123 | 0.390 | 0.384 | 0.682 | 0.155 | 0.241 | -0.446 | -0.456 |
| Ca (X_16_) | 0.278 | 0.882 | -0.217 | -0.385 | 0.108 | 0.168 | -0.002 | -0.002 |
| P (X_17_) | 0.110 | 0.350 | 0.441 | 0.784 | -0.059 | -0.092 | 0.197 | 0.202 |
| Mg (X_18_) | 0.167 | 0.531 | -0.427 | -0.759 | 0.035 | 0.054 | 0.099 | 0.101 |
| K (X_19_) | -0.043 | -0.135 | 0.491 | 0.873 | 0.100 | 0.155 | -0.136 | -0.139 |
